# Esophagitis Dissecans Superficialis Associated With Mucosal Pemphigus vulgaris: A Rare and Underrecognized Clinical Presentation

**DOI:** 10.7759/cureus.99124

**Published:** 2025-12-13

**Authors:** Amani Altaiam, Husam Saffo, Yousef Yahia

**Affiliations:** 1 Family Medicine, Primary Health Care Corporation, Doha, QAT; 2 Gastroenterology and Hepatology, Hamad Medical Corporation, Doha, QAT

**Keywords:** autoimmune dysphagia, blistering skin disease, esophagitis dissecans superficialis, immunosuppressive therapies, oral ulcer, pemphigus vulgaris, sloughing mucosa

## Abstract

Esophagitis dissecans superficialis (EDS) is an uncommon desquamative disorder most frequently associated with chemical, thermal, and medication-related injury or bullous autoimmune diseases such as pemphigus vulgaris (PV). We report a case of mucosal-dominant PV presenting with progressive oral ulceration and symptomatic EDS in the absence of cutaneous involvement. The diagnosis was confirmed by oral mucosal biopsy demonstrating suprabasal acantholysis and positive serum anti-skin immunoglobulin G (IgG) antibodies, despite nondiagnostic direct immunofluorescence. Treatment with systemic corticosteroids resulted in rapid clinical improvement. This case underscores the importance of considering mucosal-dominant PV in patients with persistent oral ulceration and gastrointestinal symptoms that fail to respond to standard initial therapy.

## Introduction

Pemphigus vulgaris (PV) is a chronic autoimmune blistering disorder caused by autoantibodies targeting desmoglein-1 and desmoglein-3, resulting in suprabasal acantholysis and intraepithelial blistering of mucous membranes and skin [1]. The oral cavity is the most commonly affected mucosal site and often represents the initial manifestation [2]. Isolated mucosal PV can be diagnostically challenging due to overlap with aphthous ulcers, mucositis, or candidiasis. Histopathologic evaluation is often critical, particularly when direct immunofluorescence findings are inconclusive [3].

Although PV occurs worldwide, the frequency is influenced by geographic location and ethnicity. Incidence rates between 0.1 and 0.5 per 100,000 people per year have been reported most frequently; however, higher rates have been documented in certain populations [4]. Inhabitants of India, Southeast Europe, and the Middle East have the greatest risk for pemphigus vulgaris [5].

Esophageal involvement in PV is underrecognized and may present as esophagitis dissecans superficialis (EDS), an uncommon condition characterized by spontaneous sloughing of the superficial esophageal squamous epithelium. A retrospective study of 21,497 esophagogastroduodenoscopy cases reported an EDS incidence of 0.03% [6]. On endoscopy, EDS appears as whitish or grayish mucosal strips or casts, typically several centimeters long, without ulceration or bleeding [7]. It can result from mucosal irritants (e.g., bisphosphonates, nonsteroidal anti-inflammatory drugs), caustic ingestion, prolonged mechanical trauma, or autoimmune blistering diseases such as PV [7,8].

This report describes a case of mucosal-dominant PV in a previously healthy male patient who developed progressive oral ulceration and gastrointestinal symptoms over almost five months without the development of the typical skin manifestations.

## Case presentation

A 45-year-old Indian male patient with a background of prediabetes and no regular medication use prior to symptom onset presented with progressive oral mucosal ulceration. He had no personal or family history of autoimmune disease. His initial symptoms began as spontaneous gingival bleeding in the absence of visible ulcers. Two to three days later, he developed a painful ulcer on the buccal mucosa.

He was initially evaluated and diagnosed with mucositis, for which he was prescribed oral amoxicillin-clavulanate for one week. Two weeks later, he developed a sore throat and hoarseness of voice. He was prescribed multiple topical and oral preparations, the names of which he could not recall, without significant improvement or a definitive diagnosis.

Over time, his oral pain progressed to the extent that he became unable to tolerate solid or liquid intake, resulting in an unintentional weight loss of approximately 10 kg in three months. He was later prescribed a course of systemic oral corticosteroids for 10 days, which led to marked clinical improvement; however, his symptoms recurred promptly after discontinuation.

Two weeks later, he developed oral thrush, so he was treated with nystatin oral suspension four times daily for 14 days. Despite antifungal therapy, his condition deteriorated over the subsequent two weeks. He was then treated with oral fluconazole 150 mg for seven days and rabeprazole 20 mg daily for one month. Despite evaluations by multiple physicians across different specialties and the use of several topical and systemic therapies, his overall condition continued to worsen. 

**Figure 1:**
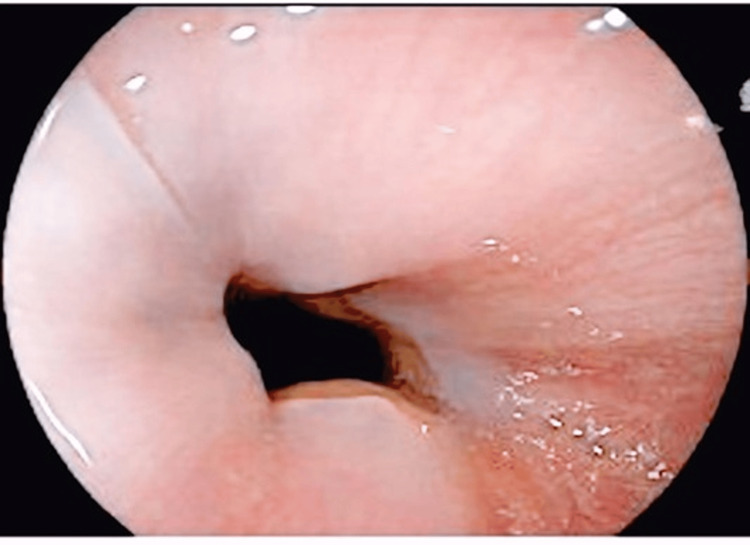
Upper endoscopic view of the distal esophagus demonstrating whitish sloughed mucosa.

**Figure 2:**
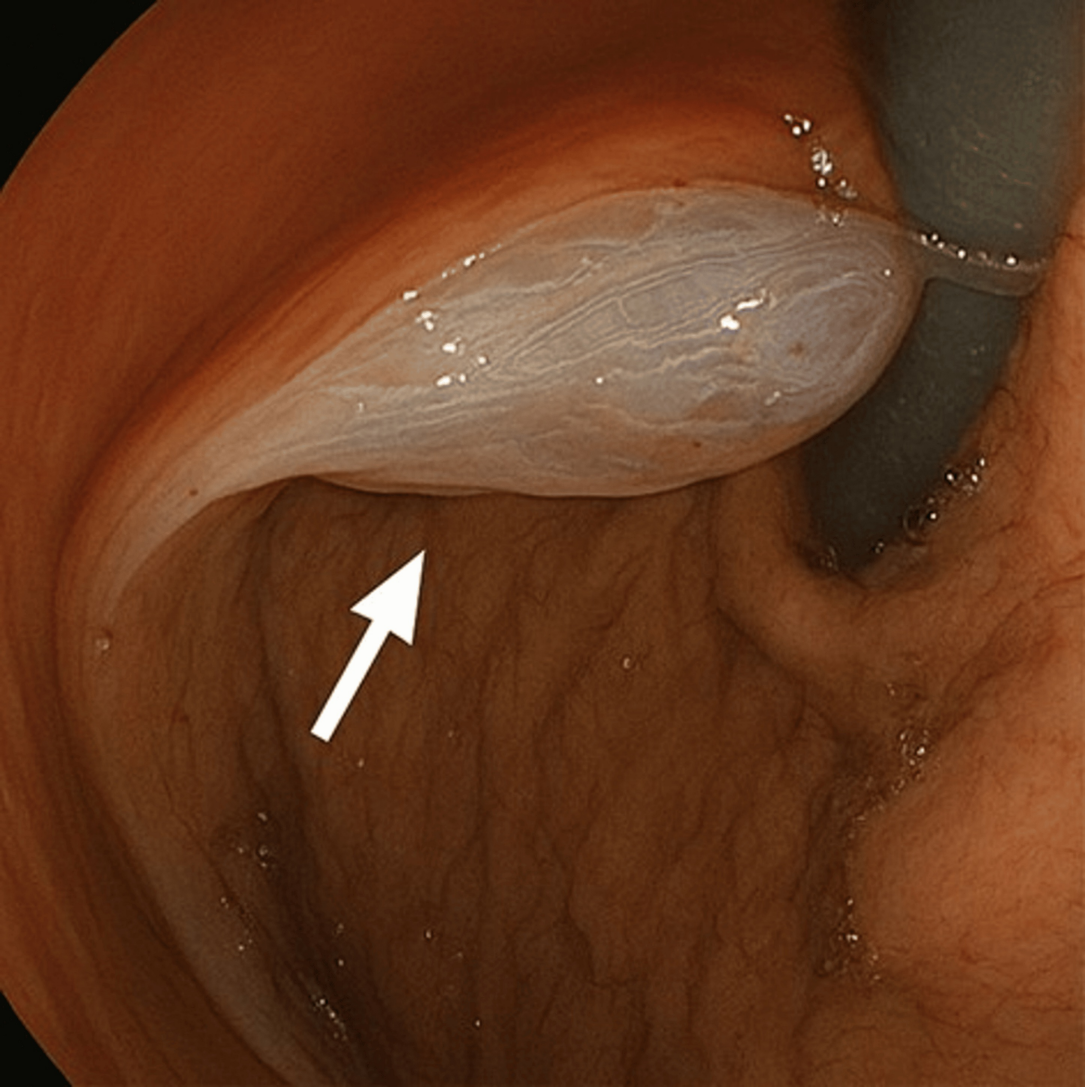
Upper endoscopic view shows sloughed esophageal mucosa in the fundus of the stomach.

**Figure 3:**
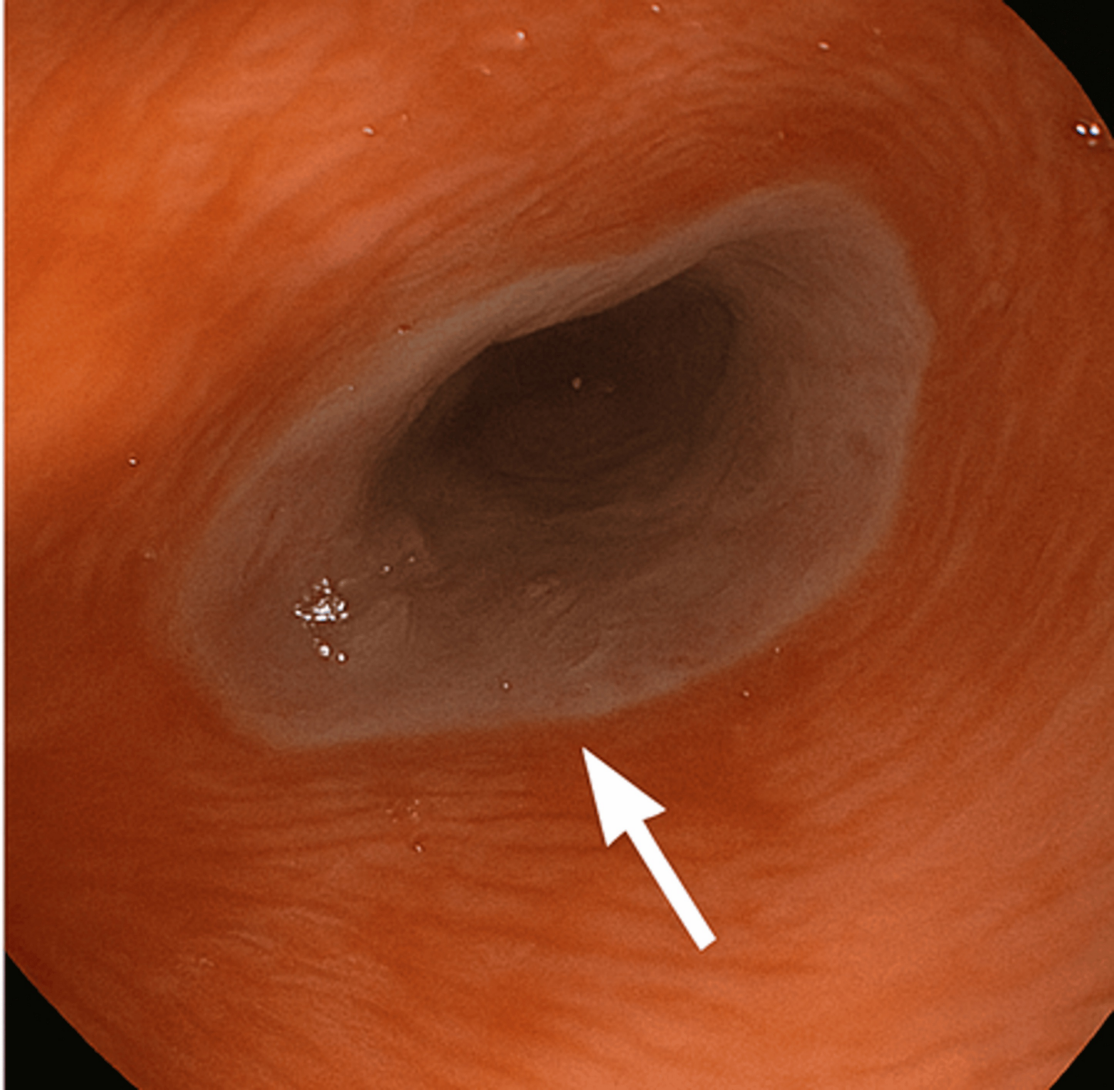
Upper endoscopic view of the distal esophagus shows a peeling mucosal flap.

He was presumed to have esophageal candidiasis, so he was referred to the gastroenterology department to undergo an upper gastrointestinal endoscopy (oesophago-gastro-duodenoscopy (OGD)). The OGD showed white desquamation of the esophageal mucosa (Figure 1). Advancing the scope revealed glove-like peeling of the superficial mucosa (Figure 2) with mild oozing but no deep tears or perforation seen (Figure 3).

After the endoscopy was done, the patient had mild epigastric pain and bloody vomitus, so he was admitted to the hospital on the same day for further investigations and management. He was given intravenous fluid and intravenous proton pump inhibitors.

All the investigations are shown below in Table 1, which were all unremarkable except for mild elevation in white blood cells and positive anti-skin antibody immunoglobulin G (IgG) and positive Quantiferon Gold test.

**Table 1:** Summary of the laboratory investigations IgG: immunoglobulin G; CTD: connective tissue disease; HIV: human immunodeficiency virus; TB: tuberculosis; CA: cancer antigen; CEA: carcinoembryonic antigen; TSH: thyroid-stimulating hormone; HLA: human leukocyte antigen; 25-OH: 25-hydroxyvitamin D

Test Category	Test	Result (Reference Range)
Complete Blood Count	Hemoglobin	Normal (13–17 g/dL)
	Platelets	Normal (150–400 ×10⁹/L)
	White Blood Cell Count	14.2 ×10⁹/L (4–11 ×10⁹/L)
Autoimmune/Immunologic Tests	Anti–skin Antibody IgG	Positive (Negative)
	Rheumatoid Factor	Negative (<14 IU/mL)
	Anti–CTD Panel	Negative
Infectious Workup	Viral Hepatitis Panel	Negative
	HIV Antibody	Negative
	QuantiFERON-TB Gold Test	Positive (Negative)
Tumor Markers	CA-125	Negative (<35 U/mL)
	CA 19-9	Negative (<37 U/mL)
	CA 15-3	Negative (<30 U/mL)
	Carcinoembryonic Antigen (CEA)	Negative (<5 ng/mL)
Other Blood Tests	Ferritin	Normal (30–400 ng/mL)
	Vitamin D (25-OH)	Normal (20–50 ng/mL)
	Vitamin B12	Normal (200–900 pg/mL)
	Thyroid-Stimulating Hormone (TSH)	Normal (0.4–4.0 mIU/L)
Imaging	Barium Swallow Study	Normal
	Chest X-ray	Normal
Genetic/HLA Testing	HLA-B51	Negative

Esophageal mucosal sloughing was extensive and endoscopically fragile; biopsy was deferred to avoid deep mucosal tearing or perforation, so a biopsy from the oral mucosal ulcers was taken after a few days and was sent for histopathology, which showed stratified squamous epithelium with suprabasilar acantholysis, leaving an attached basal layer that exhibited a characteristic “tombstone appearance.” The submucosa was remarkable for a mixed inflammatory infiltrate, composed predominantly of eosinophils, lymphocytes, and a few neutrophils. The adjacent intact squamous epithelium displayed acanthosis, spongiosis, and hyperkeratosis, consistent with reactive epithelial changes. The underlying skeletal muscle showed minimal chronic inflammation. No evidence of dysplasia or neoplastic change was identified in this biopsy. The final histopathologic diagnosis was vesiculobullous disease with features consistent with mucosal pemphigus vulgaris. Direct immunofluorescence demonstrated non-localizing positivity without classic intercellular outlining.

During his hospital admission, the dermatology team reviewed the patient and started him on oral prednisolone 20 mg daily for one week, followed by tapering to 10 mg daily, with a plan to initiate azathioprine as a steroid-sparing agent. The patient was discharged after three days of hospitalization with scheduled follow-up appointments in both the dermatology and gastroenterology clinics. He was also referred to the infectious disease outpatient clinic and was initiated on isoniazid therapy for nine months for treatment of latent tuberculosis infection. At follow-up, he demonstrated marked clinical improvement and continued regular follow-up with the dermatology team to complete his long-term management plan.

## Discussion

EDS is an uncommon desquamative disease of the esophagus characterized by sloughing of the superficial squamous mucosa, often forming sheet-like casts. Reported etiologic associations include medication-induced injury (notably bisphosphonates, potassium chloride, and nonsteroidal anti-inflammatory drugs), caustic exposure, thermal injury, autoimmune blistering disorders, and idiopathic presentations [6,9]. Histopathologic features typically include laminated necrotic squamous epithelium with minimal inflammatory response and absence of viral changes, findings that distinguish EDS from reflux, infectious, eosinophilic, or caustic esophagitis [7,10].

Esophageal involvement in PV is considered underrecognized, largely because patients are seldom investigated endoscopically unless symptoms arise. When present, PV-related acantholysis can extend to the esophageal mucosa and produce endoscopic findings indistinguishable from EDS, with broad mucosal peeling and superficial sloughing [11,12]. Early reports describing EDS in PV, including one demonstrating direct immunofluorescence positivity in esophageal tissue, support a direct autoimmune mechanism [7,8], and subsequent small series further reinforce this association [12-14].

The current case builds on existing observations linking EDS to autoimmune blistering disease but differs in several important aspects. First, this patient exhibited mucosal-dominant PV without any cutaneous lesions at the time esophageal involvement was identified. Second, his presentation involved progressive symptomatic EDS rather than incidental mucosal changes. Third, diagnostic confirmation depended on oral mucosal biopsy because esophageal sampling was deferred due to extreme mucosal fragility, an obstacle rarely addressed in earlier literature. 

This case underscores that significant esophageal involvement may occur early in the disease course, even when PV remains clinically restricted to the oral mucosa. Early endoscopic evaluation in patients with persistent odynophagia or dysphagia is therefore warranted. Management directed at controlling the underlying autoimmune activity led to symptom resolution, aligning with outcomes reported in previous PV-associated EDS cases [12-15]. 

## Conclusions

In summary, EDS should not be automatically attributed to medication or mechanical injury alone. In patients with persistent odynophagia, mucosal sloughing, and chronic oral erosions refractory to standard treatment, an underlying autoimmune blistering disorder should be considered even in the absence of skin involvement. A negative direct immunofluorescence does not exclude mucosal-dominant disease; therefore, histopathology and serologic testing remain essential. Early recognition prevents prolonged ineffective therapy and allows timely initiation of immunomodulatory treatment, leading to faster clinical recovery.
